# Emotional Reaction to Fear- and Disgust-Evoking Snakes: Sensitivity and Propensity in Snake-Fearful Respondents

**DOI:** 10.3389/fpsyg.2020.00031

**Published:** 2020-01-28

**Authors:** Silvie Rádlová, Jakub Polák, Markéta Janovcová, Kristýna Sedláčková, Šárka Peléšková, Eva Landová, Daniel Frynta

**Affiliations:** ^1^Applied Neuroscience and Neuroimaging Research Programme, National Institute of Mental Health, Klecany, Czechia; ^2^Department of Psychology, Faculty of Arts, Charles University, Prague, Czechia; ^3^Department of Zoology, Faculty of Science, Charles University, Prague, Czechia

**Keywords:** disgust, emotional response, DS-R, fear, image rating, self-reported emotion, snake phobia, SNAQ

## Abstract

This paper continues our previous study in which we examined the respondents’ reaction to two morphologically different snake stimuli categories – one evoking exclusively fear and another evoking exclusively disgust. Here we acquired Likert-type scale scores of fear and disgust evoked by the same snake stimuli by a total of 330 respondents. Moreover, we collected data about the respondents’ age, gender, education, snake fear [Snake Questionnaire (SNAQ)], and disgust propensity [Disgust Scale-Revised (DS-R)], and we analyzed the effect of these variables on the emotional scores (with special focus on snake-fearful respondents). In addition, we collected the SNAQ and DS-R scores from the respondents tested in the previous study using the rank-ordering method to directly compare the results of these two approaches. The results showed that non-fearful respondents give high scores of fear to the fear-eliciting snakes and high scores of disgust to the disgust-eliciting snakes, but they give low scores of the other emotional dimension (disgust/fear) to each. In contrast, snake-fearful respondents not only give higher fear and disgust scores to the respective snake stimuli, but they also give high scores of fear to the disgust-eliciting snakes and high scores of disgust to the fear-eliciting snakes. Both Likert-scale scores and rank-ordering data show that the clear border dividing both snake stimuli categories dissolves when evaluated by the snake-fearful respondents.

## Introduction

### Snakes as Evolutionary Threat Triggering Fear and Disgust

In the world of human ancestors, danger in many forms constantly threatening our survival was omnipresent. According to Isbell (2006, 2009), venomous snakes and large constrictors might be considered as one of the most significant predators in the primate and human evolutionary history (cf. Wheeler et al., 2011). Even though the mortality rates attributed to serpents in the prehistoric times cannot be reliably quantified due to the snake highly efficient metabolism leaving no fossil records of their prey (Greene, 1983; Hsiang et al., 2015), some circumstantial evidence suggests that the emergence of snakes must have become a strong selection pressure in the mammalian evolution (Öhman and Mineka, 2003). As a consequence of the risk presented by snakes, human ancestors have developed a complex adaptive system of interconnected fear responses manifested on the psychological, behavioral, physiological, and neural level, which, according to some authors, has been embodied in a specific brain area, the so-called module of fear (Öhman and Mineka, 2001) localized in the amygdala (Öhman, 2005; Öhman et al., 2007; for a recent critical review of the modular theory, see Coelho et al., 2019). In fact, the modular theory has drawn upon the much earlier Seligman’s (1971) concept of biological preparedness claiming that most of the clinical fears are triggered by stimuli threatening our survival in the evolutionary past. This became an alternative to the Rachman’s (1977) conditioning theory of fear learning according to which people acquire fears directly through classical conditioning, or indirectly through observation or verbally transmitted information. For a long time, there was a dispute in the literature whether snake fear is a universal inherited trait or rather a learned reaction acquired through life (for a review see, for example, Tierney and Connolly, 2013 or Kawai, 2019). However, a growing body of evidence suggests that even though fear of snakes *per se* is not innate (small infants do not fear snakes, Thrasher and LoBue, 2016, but see Hoehl et al., 2017), there is a biological predisposition to rapidly detect snakes and associate them with fear (LoBue et al., 2010).

Intensive fear of snakes has survived until today as for a majority of people, the snake is still among the most frightening animals (Davey, 1994; Polák et al., 2019b) that may trigger phobic fear in as many as 2–3% of population (Klorman et al., 1974; Klieger, 1987; Polák et al., 2016), which accounts for one of the most prevalent specific phobias (Eaton et al., 2018; cf average prevalence of any animal phobia across the world is estimated to 3.8%, Wardenaar et al., 2017). Even higher prevalence of snake phobia, despite local low abundance of snakes, was reported on a Swedish (5.5%, Fredrikson et al., 1996) or Hungarian sample (4.2%, Zsido, 2017 and 3.3%, Zsido et al., 2018). However, due to extensive species diversity with significant variability in appearance within the snake suborder (Serpentes, 3,709 species were recognized by July 2018: Uetz et al., 2019), recent studies demonstrate that besides fear, disgust is also associated with certain snakes (Janovcová et al., 2019; Polák et al., 2019b; Rádlová et al., 2019), thus it needs to be considered when studying human emotional response to these animals. Moreover, according to the influential model of disease-avoidance proposed by Matchett and Davey (1991), some animal phobias (especially those of small species) have not primarily evolved through fear of being attacked, but rather employed disgust as an adaptive mechanism protecting us from the transmission of pathogens. There is an evidence that a causal link exists between experimentally manipulated disgust and reported fear of certain animals (Webb and Davey, 1992; see also Polák et al., 2019b). It is thus reasonable to expect that snake phobia in some cases might not be driven by dysregulated fear but disgust.

### Differentiating Propensity and Sensitivity of Fear and Disgust

The latest literature on disgust suggests that it can be separated into two specific concepts, propensity and sensitivity. While the former one refers to the individual’s general tendency to respond with the emotion of disgust to various objects or situations, sensitivity is used for the secondary appraisal of disgust, i.e., how negatively the feeling of disgust itself is evaluated by the individual (van Overveld et al., 2006). So far, most of the research on disgust has been focused on propensity, while sensitivity remained overlooked. Nevertheless, it has been shown that both constructs are relevant and may be associated with several anxiety disorders (Olatunji et al., 2007; Nicholson and Barnes-Holmes, 2012), including animal phobias (Cisler et al., 2009), e.g., in predicting avoidance behavior (van Overveld et al., 2010). It is reasonable to expect that this conceptual distinction is not restricted to disgust but might be as well applied to other negative emotions such as fear. Moreover, based on the recent evidence, propensity to react with fear or disgust is associated with different brain activation pattern than sensitivity to these emotions (Mataix-Cols et al., 2008). While the former is positively correlated with activation in the attention-related (parietal and anterior cingulate cortex), and valence/arousal processing regions (orbitofrontal cortex and insula), sensitivity to fear and disgust is exclusively negatively correlated with activation in the areas involved in emotion regulation such as the medial and dorsolateral prefrontal cortex (Schäfer et al., 2009).

### Specific Psychology Profile of People With High Fear of Snakes

Differentiating propensity and sensitivity of fear and disgust is important as these constructs may be independently involved in psychopathology. For example, Vernon and Berenbaum (2008) found that fear and disgust propensity may both play their role in spider fear. Similarly, van Overveld et al. (2006) reported that while fear of blood was associated with disgust propensity and sensitivity, spider fear was correlated only with disgust propensity: according to self-report, spider-fearful respondents were more likely to react to spiders with the emotion of disgust. It is thus sensible to believe that similar results may be found in snake-fearful subjects, which differ from non-fearful controls in many aspects. For instance, it has been shown that snake-fearful respondents give more negative and extreme scores to snake stimuli when rating valence and arousal (Miltner et al., 2005) or anxiety, disgust, and pain (Lueken et al., 2011; Haberkamp et al., 2013). Their reaction time to detect snake stimuli is shorter (Öhman et al., 2001; Flykt and Caldara, 2006; Rosa et al., 2011), while their reaction time to detect a target stimulus or a change in a scene is longer when a snake picture is present as a distractor (Lipp and Waters, 2007; McGlynn et al., 2008; see also Waters and Lipp, 2008; Waters et al., 2011, for a comparison of both reaction time procedures). People with high fear of snakes also show increased cognitive interference in the Stroop test when confronted with snake-related sentences (Constantine et al., 2001; Wikström et al., 2004).

Furthermore, high-fear individuals demonstrate higher skin conductance response (SCR) when confronted with a live snake (McGlynn et al., 1973) or just a snake picture (Flykt et al., 2017), even when these are presented unconsciously (Öhman and Soares, 1994). Unconscious presentation of snakes within watched video stimuli attracts attention in form of eye saccades directed toward the areas where the snakes were presented and this effect is again more pronounced in snake-fearful participants (Rosa et al., 2014). Flykt et al. (2017) found an increased vocal response intensity and increased heartrate changes of snake-fearful participants in response to snake picture stimuli. Moreover, the neural response of snake-fearful respondents to snake movies is higher or qualitatively different: according to Lueken et al. (2011), snake-fearful respondents show higher activation within the inferior frontal operculum, middle temporal gyrus, middle cingulate gyrus, pallidum, and the cerebellum. Even their brain morphology differs as the gray matter volume in the left postcentral gyrus is increased when compared to control participants (Hilbert et al., 2015).

### Various Snake Species May Trigger Different Emotions

In short, high-fear participants change many aspects of their behavior when confronted with the feared stimuli, regardless of whether these are presented as live specimens or just moving or still pictures. All of the above-mentioned studies considered the presented stimuli as a uniform category, a general form of “snake,” or “harmless/non-venomous snake” in studies working with live animals (e.g., McGlynn et al., 1973; Klieger and Siejak, 1997). However, there are many snake species, differing in size, color, shape, texture, and also the actual dangerousness they present to humans (Kasturiratne et al., 2008; see also Rádlová et al., 2019 for a review). Previously, we have shown that human respondents are able to distinguish between various snake morphotypes and that they mostly fear vipers and allies (Landová et al., 2018; Rádlová et al., 2019) while simultaneously evaluating harmless fossorial species as not fear-eliciting at all. These findings raise a further question whether snake-fearful participants distinguish particular snake morphotypes and respond comparably to non-fearful respondents, or evaluate all snakes in general negatively.

In the previous article (Rádlová et al., 2019), we introduced two types of defined, standardized snake stimuli: one set that elicits exclusively fear and another one that elicits exclusively disgust. The stimuli were carefully standardized, reduced to differences between specific snake morphotypes but uniform in other aspects such as the background or posture. Such approach presents a great advantage because it offers well-described and characterized stimuli, free from uncertainties about the effects of other factors such as the body size, environment, background color, or lightness on the rankings given by human respondents. Still, we found that even with this reduced variability, there was a great distinction between the stimuli types as the respondents clearly distinguished and categorized each stimulus into its respective category. However, the snakes were examined using a rank-ordering method, which is optimal for analyzing differences between the stimuli but reduces differences *between the raters* – the main focus of the present study. Here we examine the relationship of fear and disgust ratings using the absolute scale (Likert-type scores), focused on differences between respondents with high and low fear of snakes.

### Aims of the Study

In this study, we focus mainly on two aspects linked to snake fear and phobias: a relative contribution of the particular emotion of fear and disgust to enhanced snake fear and generality/specificity of snake stimuli. More specifically, we aimed to test the following predictions (corresponding to alternative hypotheses) pertaining to the effect of snake fear as measured by the Snake Questionnaire (SNAQ):

1) High-fear respondents report high fear of both types of snakes (fear- and disgust-eliciting ones), thus showing increased fear propensity toward various stimuli (as opposed to high disgust propensity found in individuals with high fear of spiders van Overveld et al., 2006). This would mean that high snake fear (and consequently the SNAQ score) is strictly saturated by the fear emotion with no disgust component involved.
2) High-fear respondents report high disgust from both types of snakes (fear- and disgust-eliciting ones). In this case, anxiety provoked by snakes (as measured by the SNAQ) would in fact result from increased disgust propensity and sensitivity which would corroborate the findings of Klieger and Siejak (1997) who argued that some SNAQ items are ambiguous and may tap into disgust.
3) High-fear respondents rate all the snakes as highly fearful and disgusting at the same time, thus showing increased fear and disgust propensity. Such results would, in accordance with the study on spider fear (Vernon and Berenbaum, 2008), suggest that high fear of snakes is composed of negative evaluation in general (i.e., valence; Barrett, 2006) and that high-fear respondents (and potentially phobics) are unable to identify or distinguish between the two emotions while evaluating different snake pictures.

Additionally, we examined the effect of high disgust propensity [as measured by the Disgust Scale-Revised (DS-R)] following the same pattern. All of the above-mentioned predictions would also mean that snake-fearful subjects do not treat various snake morphotypes as distinct categories. Should the contrary be the case, the intact ability to categorize the snakes would be predicted by the following possible outcomes:

1) Although high-fear respondents compared with controls attribute higher scores to snakes, they still report higher fear from fear-eliciting than from disgust-eliciting snakes and, simultaneously, higher disgust from disgust-eliciting than from fear-eliciting snakes.
2) During the rank-ordering task, high-fear respondents do not misplace the snakes from one category into the other more often than control respondents.

## Materials and Methods

### The Stimuli

In the previous study, we introduced two sets of snake stimuli, one consisting of 40 snake pictures rated as exclusively fear-eliciting (further referred to as F snakes) and the other consisting of 40 snake pictures rated as exclusively disgust-eliciting (further referred to as D snakes; Rádlová et al., 2019; please note that the stimuli are available online for free use in research). The sets contained snakes standardized for size and placed on a blank (white) background. In the present study, both F and D snakes were mixed into one 80-picture set and presented to the respondents.

### Testing the Emotional Response of the Respondents

A total of 330 respondents (279 women, 51 men, aged 18–65; mean age 30.03; SD = 9.84) participated in the study. First, each respondent provided an informed consent, filled information about his/her age, gender, education (biological/other), and completed the SNAQ (Czech translation: Polák et al., 2016 of the original scale by Klorman et al., 1974) and DS-R (Czech translation: Polák et al., 2019a of the original scale by Haidt et al., 1994; modified by Olatunji et al., 2007). Then he/she proceeded to the task using an online web application at www.krasazvirat.cz, specially designed to test the self-reported response to animal picture stimuli on various bases, including the Likert-type scale (Likert, 1932). The instructions were to first score each stimulus (randomly presented) on a seven-point scale according to elicited fear. Then, the stimuli were presented again, this time to be scored according to elicited disgust (1 = the least fear/disgust-eliciting, 7 = the most fear/disgust-eliciting). Half of the respondents received the task in a counter-balanced order, i.e., their task was to score the set first for elicited disgust and then fear.

The Likert-type scale, which helps to acquire absolute scores for each stimulus, is a very sensitive method when considering differences among respondents. Different respondents tend to use the scale in a different way; they use the full scale only partially, give higher/lower scores to specific stimuli, etc. In contrast, the rank-ordering method, in which the respondents sort all of the stimuli in an ascending or descending order according to a specific dimension (e.g., emotion such as fear), only helps to collect relative ranks of the stimuli. Such a method is optimal for studying different patterns of the presented stimuli in general, but reduces the variability among respondents (Rádlová et al., 2019). To show the difference of the two methods in a direct comparison, we utilized the rank-ordering data of the same mixed (F-D) set from Rádlová et al. (2019), with additional data of the SNAQ and DS-R scores from 154 respondents (107 women, 48 men; mean age = 25.62; SD = 9.88). These data were collected in a very similar manner as those described above, except the evaluation method used was rank-ordering (see Marešová and Frynta, 2008; Marešová et al., 2009a for more details).

Consistently with our previous study, those participants who scored above the 75th percentile on the SNAQ (8 and higher) were then classified as “high-fear” respondents (*n* = 143). Similarly, those who scored above the 75th percentile on the DS-R (44 and higher) were classified as “high-disgust” respondents (*n* = 171). Others were classified as “low-fear” (*n* = 187) and/or “low-disgust” (*n* = 159) respondents, respectively (see Table 1 for descriptive statistics of the studied sample). By choosing the upper quartile, we could balance between an individual fear level significant enough to discover its potential effect and a statistically sufficient number of subjects within the high-fear category.

**TABLE 1:** Descriptive statistics of the study sample. **(A)** Likert-scale data: *n* = 330; high-fear group *n* = 143; high-disgust group *n* = 171; **(B)** Rank-ordering data: *n* = 172; high-fear group *n* = 44; high-disgust group = 71.

**(A) Likert-scale data**															
	**All respondents**			**High-fear respondents**			**Low-fear respondents**			**High-disgust respondents**			**Low-disgust respondents**		
	**Age**	**SNAQ**	**DS-R**	**Age**	**SNAQ**	**DS-R**	**Age**	**SNAQ**	**DS-R**	**Age**	**SNAQ**	**DS-R**	**Age**	**SNAQ**	**DS-R**
Mean	30.01	8.94	43.67	30.66	16.80	46.78	29.50	2.93	41.28	30.08	10.49	54.76	29.93	7.27	31.74
SD	9.64	8.12	14.35	9.67	6.15	14.25	9.61	2.03	14.01	9.57	8.38	9.02	9.75	7.51	8.07
Min	18	0	10	19	8	10	18	0	13	18	0	44	18	0	10
Max	65	30	95	63	30	95	65	7	87	63	30	95	65	28	43

**(B) Rank-ordering data**															
	**All respondents**			**High-fear respondents**			**Low-fear respondents**			**High-disgust respondents**			**Low-disgust respondents**		
	**Age**	**SNAQ**	**DS-R**	**Age**	**SNAQ**	**DS-R**	**Age**	**SNAQ**	**DS-R**	**Age**	**SNAQ**	**DS-R**	**Age**	**SNAQ**	**DS-R**

Mean	25.22	6.10	43.16	25.86	14.27	49.45	25.01	2.86	40.64	26.37	8.24	55.94	24.43	4.30	32.22
SD	9.43	6.10	14.64	12.16	5.44	13.01	8.33	1.82	14.55	10.95	7.07	8.96	8.16	4.45	8.28
Min	18	0	3	18	8	25	18	0	3	18	0	45	18	0	3
Max	79	27	93	74	27	93	79	7	84	74	27	93	79	24	43

### Statistical Analysis

Most data were statistically analyzed using multivariate statistics including a multiple regression and MANOVA. In these cases, effect sizes were provided as Pillai’s Trace (Pillai, 1955). In order to quantify and test congruence in species ranking provided by different respondents, we adopted a two-way, consistency, average-measures intra-class correlation (ICC; McGraw and Wong, 1996; Hallgren, 2012) computed in R (irr package). Prior to the analyses, the raw order-ranks were transformed as follows: each value minus 1 was divided by the number of evaluated species minus 1 and square-root arcsin transformed to achieve a normal distribution. A principal component analysis (PCA) was performed to visualize the multivariate structure of the data sets. Friedman test and Mann–Whitney *U*-test were used as a non-parametric alternative for variables deviating from normality (raw sores). Effect sizes for the Mann–Whitney *U*-tests were computed as normal approximation z to r (Pallant, 2007; Field, 2013). Pairwise comparisons of the means were done using the *post hoc* Nemenyi multiple comparison test.

Contribution of the explanatory variables (constrains) to the scorings and rankings of the snakes was examined using the redundancy analysis (RDA) as implemented in the R package vegan (Oksanen et al., 2017). RDA is a multivariate direct gradient method. It extracts and summarizes the variation in a set of response variables (subjective evaluation of fear and disgust evoked by snakes) that can be explained by a set of explanatory variables. Statistical significance of the gradients was confirmed by permutation tests. Calculations were performed in R (R Development Core Team, 2010) and Statistica 9.1 (StatSoft Inc, 2010).

## Results

### Likert-Scale Data

#### PC Analysis

**FIGURE 1:**
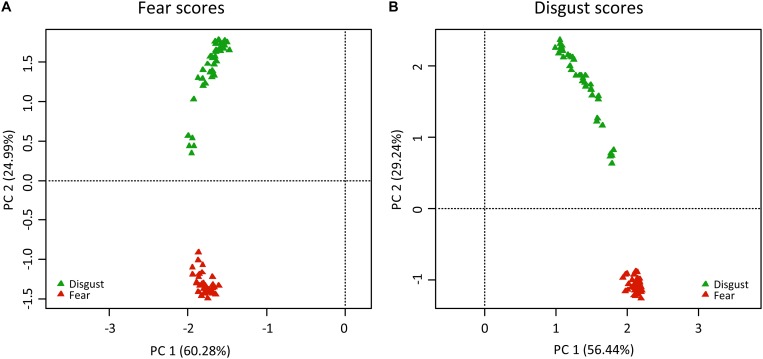
Results of PC analyses of the **(A)** fear scores and **(B)** disgust scores of snake stimuli. The colored triangles refer to the pictures of fear-eliciting snakes (red) and disgust-eliciting snakes (green). In both cases, PC2 axis contributed to the separation of the snakes into their respective categories.

Principal component analyses of the fear scores generated 80 axes, 12 of which were of an eigenvalue higher than 1. The most variability was explained by the first two axes: PC1 explained 60.28% and PC2 explained 24.99% of the full variability (see Figures 1A,B). The second axis clearly separated the stimuli into the two groups of fear-evoking and disgust-evoking snakes. Very similar results were found when analyzing the disgust scores: 80 PC axes, the eigenvalues of 13 of which were higher than 1; PC explained 56.44% and PC2 29.24% of the full variability. Again, the PC2 axes separated the stimuli into the two groups. In both cases, the rest of the axes (3–80) explained <1% variability each.

#### Agreement Among Respondents

Results revealed considerable congruence among the respondents in fear scores. Although reliability of the individual rankings (Hallgren, 2012) was only moderate (ICC = 0.416, *p* < 0.0001), ICC for the average-measures was in an excellent range: ICC = 0.996, *p* < 0.0001 (Shrout and Fleiss, 1979; Cicchetti, 1994). These results indicate that there was a high degree of agreement within the group of respondents and suggest that the snake stimuli were rated similarly in terms of evoked fear. Similarly, a high agreement for the average measures was found when analyzing the disgust scores (ICC = 0.982, *p* < 0.0001), although the single measures agreement was somewhat lower (ICC = 0.144, *p* < 0.0001).

#### Variability Among Respondents

We performed a multivariate multiple regression (Type II MANOVA tests) to analyze the effect of age, gender, SNAQ and DS-R scores, education, and order of the task on the scores. In the case of fear scores, only the SNAQ (Pillai’s Trace = 0.666, *F*_1_,_323_ = 6.09, *p* < 0.0001), education (Pillai’s Trace = 0.382, *F*_1_,_323_ = 1.887, *p* = 0.0001), and the task order (Pillai’s Trace = 0.406, *F*_1_,_323_ = 2.088, *p* < 0.0001) were significant. In the case of disgust scores, significant predictors were age (Pillai’s Trace = 0.316, *F*_1_,_323_ = 1.411, *p* = 0.0244), SNAQ (Pillai’s Trace = 0.739, *F*_1_,_323_ = 8.64, *p* < 0.0001), education (Pillai’s Trace = 0.379, *F*_1_,_323_ = 1.863, *p* < 0.0001), and the task order (Pillai’s Trace = 0.308, *F*_1_,_323_ = 1.359, *p* = 0.0396). To identify the species that substantially contributed to these differences, we performed Mann–Whitney *U*-tests comparing the raw ranks of each species in low/high fear respondents, biologists/non-biologists, and respondents first scoring fear/disgust, respectively; the levels of significance were Bonferroni-corrected. The differences in both fear and disgust scores of low versus high fear respondents were strongly significant (*p* < 0.0001) in all cases (all snake species; for more details and effect sizes, see Supplementary Material 1). In the case of fear scores, the education affected only the disgust-evoking snakes, which were scored as less fear-evoking by biologists (all *p* < 0.0001). In the case of disgust scores, biologists scored the majority of fear-evoking snakes (30 out of 40) as less disgusting than did the non-biologists, and also three disgust-evoking snakes ware rated as less disgusting. Additionally, the respondents who first evaluated the stimuli according to fear scored 26 of the disgusting snakes as less fear-evoking and three of the fear-evoking snakes as more fear-evoking. For more detailed statistics including effect sizes computed as normal approximation z to r (Pallant, 2007; Field, 2013), see Supplementary Material 1. In the case of disgust scores, no snake was significant. We have also performed the same analysis for women only, but this approach yielded comparable results (for more details, please see Supplementary Material 2).

It is possible that the effect of gender was not significant because the gender ratio in our sample was very unbalanced (51 men, 279 women). Because of that (and to control for the effect of SNAQ, which was the strongest predictor, see also the RDA analyses below), we randomly selected 51 women from the sample with the corresponding SNAQ scores, pooled them together, and re-analyzed the data. In the case of fear scores, significant predictors were the SNAQ (Pillai’s Trace = 0.935, *F*_1_,_99_ = 3.581, *p* < 0.0011) and education (Pillai’s Trace = 0.911, *F*_1_,_99_ = 1.863, *p* = 0.0102); see Supplementary Material 2 for more details. The gender appeared as a single significant predictor only in the case of disgust scores (Pillai’s Trace = 0.887, *F*_1_,_99_ = 1.957, *p* = 0.0456). However, a univariate analysis of the disgust scores revealed that the effect of gender was significant in neither case (snake), and these results were confirmed by Mann–Whitney *U*-tests (Bonferroni corrected). This suggests that no strong effect of certain species contributes to the results, but rather that it is constructed by a combination of a number of small effects. This could be, however, also an artifact of the statistical method.

**FIGURE 2:**
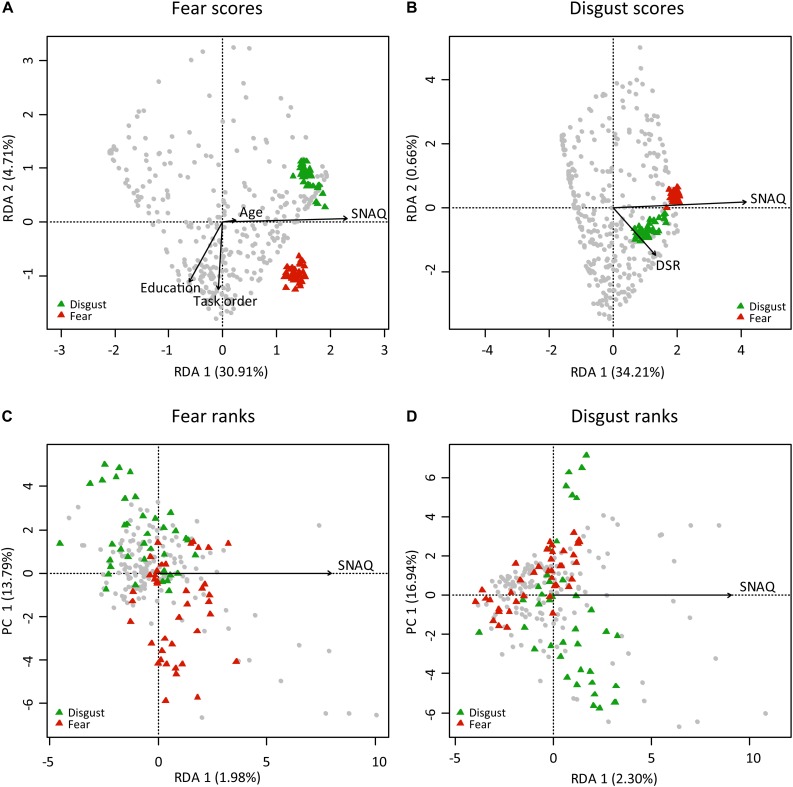
Redundancy analysis (RDA) of the respondents characteristics determining their ratings [scores **(A,B)** and rank-orderings **(C,D)**] of fear and disgust elicited by snake stimuli. The colored triangles refer to the pictures of fear-eliciting snakes (red) and disgust-eliciting snakes (green). In all cases, the effect of SNAQ scores was a significant predictor (ANOVA). However, in the case of Likert-type scoring **(A,B)**, the effect was much higher than in the case of the rank-ordering, because the latter method uses relative ranks and minimizes variability among the respondents. It is thus more suitable for analyses of variability among the stimuli.

A redundancy analysis confirmed the results of the regressions. We utilized the automatic model-building feature based on both Akaike criterion (but with permutation tests) and on permutation *P*-values. In the case of fear scores, both methods agreed on the inclusion of the following variables into the reduced model: SNAQ scores, age, education, and order of the task. The reduced model has generated four constrained axes that explained 35.79% of the full variability. Sequential “Type I” ANOVA (*n* permutations = 10,000) further revealed that the effect of SNAQ scores (*F*_1_,_325_ = 153.151, *p* < 0.0001), education (*F*_1_,_325_ = 6.756, *p* = 0.0012), and order of the task (*F*_1_,_325_ = 18.560, *p* < 0.0001) on fear scores were significant. In the case of disgust scores, only the SNAQ (*F*_1_,_327_ = 169.627, *p* < 0.0001) and DS-R (*F*_1_,_327_ = 5.440, *p* = 0.0046) scores were significant. Therefore, we have also tried to recalculate the analysis using scores on three individual DS-R subscales known as core, animal reminder, and contamination-based disgust instead of DS-R total scores. The reduced model revealed again the effect of SNAQ scores (*F*_1_,_327_ = 171.817, *p* < 0.0001), but out of the three disgust subscales, only core disgust has proved significant (*F*_1_,_327_ = 8.331, *p* = 0.0003). Interestingly, this model better explained the full variability than the one using DS-R total scores (35.73 vs 34.87%, respectively). For more details, see Table 2 and Figures 2A,B.

**TABLE 2:** PCA and RDA results of the fear and disgust scores and ranks.

	**Fear scores**	**Disgust scores**	**Fear ranks**	**Disgust ranks**
**% Explained**				
Constrained%	35.79%	34.87%	2.34%	2.30%
No. RD axes	4	2	1	1
RD1	30.91%	34.21%	2.34%	2.30%
RD2	4.71%	0.66%	–	–
**Eigenvalues**				
RD1	92.61	118.77	221.83	365.02
RD2	14.11	2.28	–	–
PC1	90.89	101.17	0.38	0.68
PC2	58.40	75.74	0.33	0.45
**ANOVA *p*-values**				
SNAQ	<0.0001	<0.0001	0.0005	0.0003
DS-R	–	0.0046	–	–
Task order	<0.0001	–	–	–
Education	0.0012	–	–	–
Age	0.0605	–	–	–

#### Analysis of Mean Scores

Next, we analyzed the effect of dimension (i.e., the emotion according to which the set was scored) and set (D vs F snakes) in relation to snake fear. For each respondent within the high-fear and low-fear category, we computed mean fear scores (fear) and mean disgust scores (disgust) separately for disgust-evoking snakes (D) and fear-evoking snakes (F; these variables are further referred to as fear-D, fear-F, disgust-D, and disgust-F). Friedman tests revealed that the effect of combination of dimension and set on mean scores was highly significant for both high-fear subjects (Friedman chi-squared_3_ = 123.57, *p* < 0.0001) and low-fear subjects (Friedman chi-squared_3_ = 234.94, *p* < 0.0001). Furthermore, we performed pairwise comparisons within each respondents’ group using the *post hoc* Nemenyi test. Within the low-fear subjects, all comparisons were highly significant (all *p* < 0.0001) except for disgust-D vs fear-F, which was significant at the *p* = 0.037 level, and disgust-F vs fear-D, which was not significant. Within the high-fear subjects, all comparisons were highly significant (all *p* < 0.0001) except for disgust-F vs fear-D and disgust-D vs disgust-F, which were not significant.

**FIGURE 3:**
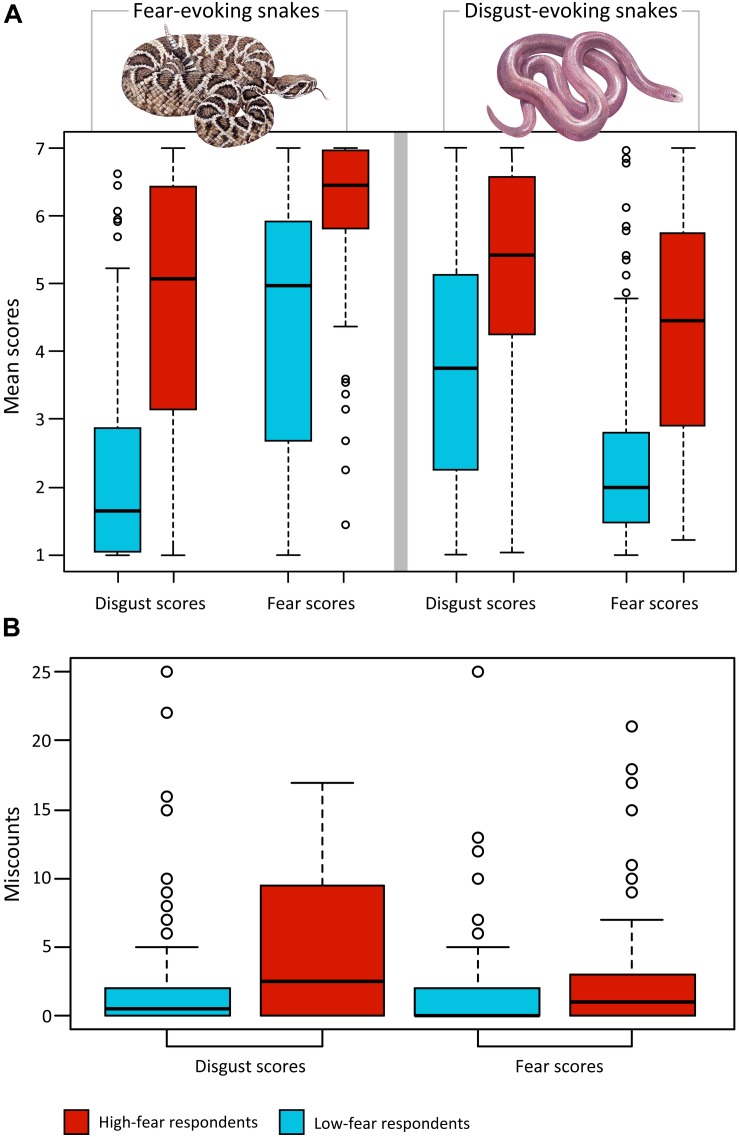
Comparison of snake ratings of high-fear and low-fear respondents. **(A)** The effect of combination of dimension (fear and disgust scores) and set (fear-evoking and disgust-evoking stimuli) on mean scores was highly significant for both high-fear subjects and low-fear subjects (both *p* < 0.0001). A *post hoc* Nemenyi test revealed that within the low-fear subjects, out of six comparisons, only one was not significant (disgust-F vs fear-D, the capital letter marks stimulus category). In the case of the high-fear subjects, disgust-F vs fear-D and disgust-D vs disgust-F were not significant. All comparisons outside of the high-fear and low-fear groups were highly significant (all *p*s < 0.0001). **(B)** Analysis of miscounts: SNAQ scores affected the number of miscounts (i.e., how many times a respondent misplaced snake from one category into the other category; *p* = 0.0005); the high-fear subjects misplaced the snake out of its category more often than low-fear subjects, regardless of the dimension of rank-ordering.

We then analyzed the mean scores outside of the groups using the Mann–Whitney *U*-tests, which revealed that all comparisons were highly significant (all *p* < 0.0001); disgust-F: *U* = 3484.5, *r*^2^ = 0.402; disgust-D: *U* = 7052.5, *r*^2^ = 0.164; fear-F: *U* = 4877.0, *r*^2^ = 0.297; fear-D: *U* = 4550.5, *r*^2^ = 0.320). For a graphical summary, see Figure 3A.

### Rank-Ordering Data

#### PC Analysis

Principal component analyses of the fear and disgust ranks were also very similar to each other: each analysis generated 79 axes, none of which had the eigenvalue higher than 1. The first and second axes explained 12.55 and 11.31% of the full variability in the case of fear ranks and 16.99 and 12.55% of the full variability in the case of disgust ranks.

#### Agreement Among the Respondents

In the case of the rank-ordering data, agreement of the respondents was very high in both fear (ICC = 0.997 for average-measure, 0.683 for single-measure) and disgust (ICC = 0.996 for average-measure, 0.606 for single-measure) evaluations (all *p* < 0.001). These results are in agreement with the Kendall’s coefficients of concordance as provided in Rádlová et al. (2019) and show that the respondents were reliably able to sort the snakes into their respective groups (i.e., sorted most of the fear-evoking snakes as the first most fear-evoking when ranking the set according to the evoked fear, etc.).

#### Variability Among Respondents

In the analyses, we used data about the age, gender, education, and task order from Rádlová et al. (2019) as well as the newly collected SNAQ and DS-R scores. A multivariate multiple regression analysis of the fear ranks revealed that only the effect of age (Pillai’s Trace = 0,659, *F*_1,78_ = 1.734, *p* = 0.0100) and task order were significant (Pillai’s Trace = 0.628, *F*_1,78_ = 1.516, *p* = 0.0389). Mann–Whitney *U*-tests revealed that after Bonferonni correction, the significant effect of task order remained in only one snake, the *Austrotyphlops diversus*, which was rated as more fear-eliciting by the respondents that were first to rank the pictures according to elicited disgust (*U* = 1955.5, *r*^2^ = 0.085, *p* = 0.0003). A corresponding regression analysis of the effect of age, gender, SNAQ and DS-R scores, education, and order of the task on the disgust ranks revealed no significant effect of any of these factors.

A redundancy analysis of the fear and disgust rankings did not confirm the regressions. Only the SNAQ scores significantly explained the rankings, but the effect was very small: the constrained axes explained 1.98% in the case of fear ranks (ANOVA: *F*_1_,_152_ = 3.062, *p* = 0.0005) and 2.30% in the case of disgust ranks (ANOVA: *F*_1_,_152_ = 3.574, *p* = 0.0003; see Table 2 and Figures 2C,D).

#### Analysis of Miscounts

Although the rank-ordering task required only sorting the pictures according to the given dimension, the respondents were able to unconsciously categorize the snakes by clustering together the 40 fear-evoking snakes as the top 40 fear-evoking ones and the 40 disgust-evoking snakes as the bottom 40 fear-evoking ones (Rádlová et al., 2019). In the case of disgust dimension, the results were similar but opposite. Whenever the respondent misplaced a snake outside of its place (category), we counted this as a miscount. The total number of miscounts was collected for each respondent and further analyzed. In the subsequent glm analysis, we examined the effect of dimension, SNAQ score, and their interaction to the number of miscounts (quasipoisson model). The results revealed that only the SNAQ scores affected the number of miscounts (*p* = 0.0005); the high-fear subjects misplaced a snake out of its category more often than low-fear subjects, regardless of the dimension of rank-ordering (Figure 3B).

## Discussion

### Generality/Specificity of Snake Stimuli

In the previous study (Rádlová et al., 2019), although uninstructed to do so, the respondents clearly assorted the mixed set of fear- and disgust-eliciting snakes into their respective, distinct categories. And although such results suggested that the fear-eliciting snakes do not elicit any disgust and the disgust-eliciting snakes do not elicit any fear, one could not be entirely sure as the evaluation was done using a relative scale. In this study, we confirmed that this was true for low-fear subjects by asking the participants to score the same set of mixed F-D snake stimuli on an absolute scale. The results showed that the fear-eliciting snakes received significantly much higher scores of fear and lower scores of disgust than the disgust-eliciting snakes, for which the opposite was true. Moreover, the fear scores of the D-snakes and disgust scores of the F-snakes did not significantly differ from each other – both were very low and indicated that the F-snakes elicited no disgust and D-snakes elicited no fear. In comparison, the same scores given by the high-fear respondents also did not differ significantly from each other, but both were significantly higher than those of the low-fear respondents. In other words, high-fear respondents find snakes that usually (i.e., in people with normative fear) evoke no disgust disgusting and snakes that usually evoke no fear fear-evoking (Figure 3). These results suggest that both fear and disgust propensity are involved in high snake fear (and possibly phobia). Moreover, disgust elicited by the F and D snakes did not significantly differ, which points out that the high-fear respondents do not distinguish between the two categories of snake types when considering their emotional effect. However, they still distinguish the F snakes as significantly more fear-eliciting than the D-snakes.

Analysis of the number of miscounts from the rank-ordering data also confirmed that the high-fear respondents partially lose the ability to distinguish between the two snake categories as they misplaced the snakes from one category into the other category significantly more often than the low-fear subjects.

### Contribution of Fear and Disgust to Snake Fear

Klieger and Siejak (1997) argue that the SNAQ is not a good measurement of snake fear because it is strongly biased by false positives. These authors found out that respondents undergoing a behavioral approach test (BAT) with a live snake often facially expressed disgust, and performed a study examining the relationship between SNAQ scores and disgust determined using various measurements. They showed that many respondents with high SNAQ scores actually approached the live snake during the BAT with no avoidance and their results suggested that it was either because the SNAQ was affected by disgust of snakes or because fear and disgust might be inseparably connected in this case.

In our study, we asked the respondents to rate both fear and disgust of snakes and compared these data with their SNAQ and DS-R scores. The DS-R scores seem to be a good measurement of disgust propensity because it affected only the scores of disgust of snakes. However, the SNAQ scores affected scoring and ranking of both fear and disgust and thus it is in agreement with Klieger and Siejak’s (1997) hypothesis that the SNAQ reflects the two emotions elicited by snakes (see also Wiens et al., 2008, where disgust sensitivity correlated with the SNAQ score). Moreover, our results show that when scoring and rank-ordering the snake pictures according to perceived disgust, the high-fear (high-SNAQ) respondents are not able to distinguish between the particular snake morphotypes. This may be due to the fact that high-fear respondents feel strong disgust not only from the disgust-eliciting snakes, but also from the fear-eliciting ones (vipers), otherwise rated as not-disgusting at all by low-fear respondents. Similar results were found in Polák et al. (2019b), where respondents with high SNAQ scores rated both the venomous viper and harmless grass snake as strongly fear- and disgust-eliciting. In comparison, low-SNAQ respondents only rated the viper as fear-eliciting.

Another explanation of this phenomenon is that the high-fear respondents cannot distinguish the emotions and only evaluate the snakes according to negative valence (Barrett, 2006; Barrett and Wager, 2006). It may be possible that simply seeing the snake stimuli made the high-fear respondents feel miserable (cf. core affect, Russell and Barrett, 1999), which in turn affected the overall evaluation negatively, but it was still hard for the respondents to assign a particular emotional label to a single snake.

### Are Likert-Scale and Rank-Ordering Data Comparable in Evaluation of Perceived Snake Fear and Disgust?

In this paper, we tested the self-reported emotional reactions toward snake pictures using the Likert scale, and we compared the results with those of the rank-ordering scale used in Rádlová et al. (2019). Each method has its advantages and disadvantages, and should be thus used in purposely designed experiments. The absolute Likert-type scale is sensitive to the differences between respondents and is better to be used in experiments in which differences between two groups of respondents, e.g., low- and high-fear respondents like in this paper, are the main focus of interest. However, when a study uses a block of similar stimuli that is treated and measured as a single condition (e.g., studies involving eye-tracking, reaction time, EEG, fMRI), a thorough examination of the variability among the stimuli is needed to ensure that each stimulus within the block has the same properties of interest. Failing to do so may lead to high noise, skewed results, or even a wrong interpretation. And for this, the rank-ordering method is optimal as it maximizes the variability among the stimuli (Rádlová et al., 2019). However, relative ranks minimize the variability among respondents and are thus unsuitable for studies focused on the factors behind respondents’ variability. Here we compare both methods to further illustrate the impact of each of them on the results of respondents’ characteristics including the age, gender, SNAQ and DS-R scores, education, and order of the task.

The regression analyses of the rank-ordering data revealed no effect of the respondents’ variables on the disgust ranks, and also revealed only the effect of age and order of the task on the fear ranks. However, these effects were not strong enough to survive a different type of analysis: the RDA only revealed the effect of SNAQ in both cases, and it was very small (1.81 and 2.34%). These results point out that there is only a little difference between the respondents on the relative order of the ranked stimuli. Similar results were presented in other studies using the rank-ordering method and analyzing not only snake stimuli (Marešová and Frynta, 2008; Marešová et al., 2009a, b; Matchett and Davey, 1991; Frynta et al., 2011; Landová et al., 2012; Ptáčková et al., 2017), but also other animals (Frynta et al., 2009, 2010, 2013; Lišková et al., 2015). In other words, if a viper is ranked as more fear-evoking than a blind snake, the order stays the same (Landová et al., 2018), even if a high-fear respondent finds both snakes much more fear-evoking than a low-fear respondent. Similarly, in our study, all respondents regardless of age, gender, or order of the task ranked the D-snakes as very disgusting (grouping them together) and the F-snakes as very fear-evoking. However, although the effect of the SNAQ score is small, it still leads to significantly larger number of miscounts in high-fear respondents who tend to rank the F-snakes as more disgusting.

When compared to the absolute scale data, the analyses revealed much higher effect of the respondents’ characteristics on both fear and disgust scores: according to the RDA, SNAQ, age, education, and order of the task together explained as much as 35.79% of the full variability of the fear scores and the SNAQ and DS-R together explained 34.87% of the variability of disgust scores. The SNAQ scores, in both cases building the RD1 axis, had the highest effect (Figure 2): the high-fear respondents scored all of the snake stimuli much higher than the low-fear respondents. Moreover, respondents with biological education scored the F-snakes as less disgusting and the D-snakes as less fear-evoking. In comparison, Prokop and Tunnicliffe (2008) found the effect of knowledge only on the attitudes toward non-feared animals (bats), but not phobia-related animals such as spiders. The authors argued that public awareness is not enough to improve attitudes toward animals that were associated with danger in human evolutionary history, and Tomažič (2011) found that knowledge of snakes does not affect fear of these animals. However, biologically educated people do not necessarily need to have a higher knowledge than non-biologists (Tomažič, 2011), and it is thus possible that it was rather their higher experience with live animals that affected the scores (Ballouard et al., 2012). This has been recently corroborated by Coelho et al. (in prep.) who reported that people having more experience with snakes and those bitten by a snake show lower snake fear.

When taken together, the results of both methods show not only that the high-fear respondents give overall higher scores to all of the snake stimuli, but also that they treat the D-snakes and F-snakes differently: they tend to rank the F-snakes as more disgusting and the D-snakes as more fear-eliciting than the low-fear respondents do. This difference between snake-fearful subjects and controls might form a new pictorial assessment of snake phobia. Moreover, we found that not only fear but also disgust contributes to high snake fear. Thus, a therapy focused on both of these emotions, not just fear, could lead to better treatment outcomes.

## Conclusion

The results of this study confirmed those reported in Rádlová et al. (2019): the fear-eliciting (F) snakes received high scores of fear and low scores of disgust, while the disgust-eliciting (D) snakes received high scores of disgust and low scores of fear. Thus, human respondents are apparently able to distinguish these characteristic snake morphotypes (and possibly many others) and respond accordingly to each. Additionally, we found that high-fear respondents gave high scores of both fear and disgust to all snakes, and also miscounted snakes within each category more often than low-fear respondents, partially dissolving the boundaries of both categories. Thus, while it is natural to fear dangerous snakes, high-fear (or phobic) respondents do not only experience more intense fear (and/or disgust), they also attribute a strong emotional charge to stimuli otherwise considered safe. This might suggest that both sensitivity (i.e., high-fear respondents report more intense fear of fear-eliciting snakes than low-fear respondents) and propensity (i.e., high- vs. low-fear respondents are more likely to rate fear-eliciting snakes as highly disgusting and disgust-eliciting snakes as highly frightening) play a role in acquisition and maintenance of snake fear.

Finally, it is noteworthy that our results might have important clinical implications. So far, one of the most recommended therapeutic interventions in snake phobia, a cognitive behavioral therapy, is mostly focused on effective fear management. However, our data provide evidence that individuals with high fear of snakes experience not only elevated fear, but disgust as well, which is partly in agreement with the disease-avoidance model by Matchett and Davey (1991). Therefore, shifting focus by incorporating the disgust propensity and sensitivity component into the treatment model for snake phobics might potentially lead to improved therapeutic outcomes.

## Limitations of the Study

One of the limitations of the study emerges from the data collection method. It is well known that even though online surveys may collect extensive amount of data in a short time, these are less reliable than data from research conducted in contact with individual respondents that allows for more clarifications or corrections. Furthermore, self-reports may be often biased due to demand characteristics pertaining to the individual tendency to comply with the researcher’s expectations or attempts to present oneself in a better light based on social expectations. Therefore, within a distant and anonymous setting of online testing, the subjects with various motives are more likely to provide distorted or randomly fabricated answers that are difficult to be identified.

The second limitation may be related to the unbalanced gender ratio. Sex differences in fear and disgust (not only of snakes) is a trend continuously demonstrated throughout the psychology research. This was also the reason we had the unbalanced gender ratio within our sample, because we selected our respondents *based* on the SNAQ scores. However, when we balanced the gender in a sub-sample based on the SNAQ scores, there was no effect of gender on fear scores of the snake stimuli, and only weak (not very robust) effect of gender on disgust scores. These results mean that even though women have higher SNAQ scores and/or fear snakes more often, women and men with the same SNAQ scores rate the snake stimuli similarly. This is a very important result for further studies in which fear is the main focus, e.g., specific snake phobia (there are also male phobics and they do not differ in their fear pattern from female phobics).

## Data Availability Statement

The datasets generated and/or analyzed during the current study are available in the Mendeley repository: doi: 10.17632/ksy5z9z3fh.2.

## Ethics Statement

This study was reviewed and approved by the Institutional Review Board (IRB), Faculty of Science, Charles University, approval n. 2013/7, and by the Ethical Committee of the National Institute of Mental Health n. 55/16. The patients/participants provided their written informed consent to participate in this study.

## Author Contributions

DF and EL contributed to the conception and design of the study. MJ, KS, ŠP, SR, and JP organized the database and performed the research. SR and DF performed the statistical analysis. SR wrote the first draft of the manuscript. DF and JP wrote sections of the manuscript. All authors contributed to the manuscript revision, read and approved the submitted version.

## Conflict of Interest

The authors declare that the research was conducted in the absence of any commercial or financial relationships that could be construed as a potential conflict of interest.
